# Growth after hematopoietic stem cell transplantation in children with acute myeloid leukemia

**DOI:** 10.1186/1687-9856-2013-S1-P44

**Published:** 2013-10-03

**Authors:** Seung Joon Chung, Jieun Lee, Min Jae Kang, Choong Ho Shin, Sei Won Yang

**Affiliations:** 1 Department of Pediatrics, Seoul National University Hospital, Seoul, Korea

## 

Previous studies have shown that hematopoietic stem cell transplantation (HSCT) may result in growth impairment. Disease type, differences in treatment before HSCT and treatment duration before HSCT can affect growth after HSCT and act as confounding variables. By contrast, acute myeloid leukemia (AML) patients receive HSCT during their first remission and are not treated by steroids during their relatively short period of induction therapy time. The purpose of this study was to evaluate 5 years growth after HSCT and to find factors influencing final adult height (FAH) in childhood AML patients.

Among 97 AML patients whom received HSCT in Seoul National University Hospital, we report 24 patients whose puberty began at least 3 years after HSCT and 19 patients who reached FAH without relapse. Medical records were retrospectively reviewed. Summary measure analysis was used to evaluate for 5 year growth after HSCT and to find statistical differences between factors. Univariate and multivariate regression analysis was performed to find factors influencing FAH.

Five years growth after HSCT: Patients received HSCT at 4.2 years of age. Six patients received radiotherapy (RT) and chronic GVHD (cGVHD) were in 4 patients. History of RT and cGVHD significantly impaired the first 5 years growth after HSCT. But cGVHD seems to influence to only the first 2 years growth after HSCT. Age at HSCT, gender and history of steroid use were not significantly affected 5 years growth after HSCT.

Final adult height after HSCT: Patients received HSCT at 10.1 years of age. Four patients received RT. In patients reached FAH without relapse after HSCT, only history of RT significantly reduced FAH. Age at HSCT, gender and history of steroid use were not significantly affected FAH.

Growth impairment after HSCT in AML patients might be occurred. But without RT history, growth impairment seems to be temporary and improve by catch-up growth. HSCT with conditioning regimen consists of only chemotherapy thought to be not to significantly decrease FAH. So the growth hormone treatment is seems to be not needed in non-RT patients. But in patients who received RT, catch-up growth will not be shown and eventually attain reduced FAH.

